# TittaLSL: A toolbox for creating networked eye-tracking experiments in Python and MATLAB with Tobii eye trackers

**DOI:** 10.3758/s13428-025-02714-2

**Published:** 2025-06-04

**Authors:** Diederick C. Niehorster, Marcus Nyström

**Affiliations:** 1 Lund University Humanities Lab, Lund University, Lund, Sweden; 2 Department of Psychology, Lund University, Lund, Sweden

**Keywords:** Eye tracking, Tobii, Toolbox, Network, Multiple participants, Joint attention, Hyperscanning, Lab streaming layer

## Abstract

Studying the behavior of multiple participants using networked eye-tracking setups is of increasing interest to researchers. However, to conduct such studies, researchers have had to create complicated ad hoc solutions for streaming gaze over a local network. Here we present TittaLSL, a toolbox that enables creating networked multi-participant experiments using Tobii eye trackers with minimal programming effort. An evaluation using 600-Hz gaze streams sent between 15 different eye-tracking stations revealed that the end-to-end latency, including the eye tracker’s gaze estimation processes, achieved by TittaLSL was 3.05 ms. This was only 0.10 ms longer than when gaze samples were received from a locally connected eye tracker. We think that these latencies are low enough that TittaLSL is suitable for the majority of networked eye-tracking experiments, even when the gaze needs to be shown in real time.

## Introduction

Eye tracking is used to record where people look and how they move their eyes (Holmqvist et al., 2011), and finds application in a wide array of research fields as well as industry (e.g., Duchowski, 2002; Holmqvist & Andersson, 2017; Hessels et al., 2024). While eye tracking is often performed on single participants, there is a growing interest in using eye trackers to study the gaze behavior of multiple co-present or interacting participants (Wohltjen & Wheatley, 2024; Pfeiffer et al., 2013). These studies often use hyperscanning approaches, that is eye tracking multiple participants at the same time, to probe topics such as joint attention (Caruana et al., 2017; Yu & Smith, 2017, 2013; D’Angelo & Begel, 2017; Vertegaal et al., 1997), the effect of social presence on task performance (Oliva et al., 2017; Strukelj et al., 2016; Song et al., 2023), conversation (Capozzi & Ristic, 2022; Mayrand et al., 2023), collaboration (Raeithel & Velichkovsky, 1995; Velichkovsky, 1995; Brennan et al., 2008; Neider et al., 2010; Messmer et al., 2017; Niehorster et al., 2019; Siirtola et al., 2019; Wahn et al., 2020; Hessels et al., 2023; Atweh & Riggs, 2024; Hessels et al., 2025; see D’Angelo & Schneider, 2021; Wahn & Schmitz, 2023, for reviews), and educational applications (Špakov et al., 2019; Schneider et al., 2016, 2018). In a specific subclass of these experiments, researchers have linked multiple eye trackers together over a computer network, allowing participants to see each other’s gaze location on a screen (e.g., Brennan et al., 2008; Niehorster et al., 2019) or in settings where participants could move freely (e.g., Sung et al., 2021; Lee et al., 2017; Jing et al., 2021). Experiments using such gaze sharing are of special interest since they allow both closely monitoring and also manipulating the important communicative and monitoring roles played by gaze (Hessels, 2020; Gobel et al., 2015).

Many tools exist for locally interfacing with eye trackers (e.g., Cornelissen et al., 2002; Dalmaijer et al., 2014; De Tommaso & Wykowska, 2019; Niehorster & Nyström, 2020, Niehorster et al., 2020a, 2024b; see Niehorster et al., 2025, for an overview). However, until now, there has not been an easy-to-use toolbox for streaming gaze data over the network, allowing straightforward implementation of networked eye-tracking experiments. Previous experiments, such as Brennan et al. (2008) and Niehorster et al. (2019) used custom solutions (see D’Angelo & Schneider, 2021, for a discussion), while Nyström et al. (2017) provide a minimal working example of a toolkit for SMI eye trackers and Python that left a lot of the complexity of dealing with multiple networked eye tracker data sources to the end user. Similarly, while SR Research EyeLink devices allow sending data to any computer over the network, handling the data streams is left to the user. The lack of an easy-to-use tool for handling networked eye tracker data sources entails a significant barrier to creating studies requiring real-time gaze streaming even for researchers who have good general knowledge of programming. As D’Angelo and Schneider (2021) said, “This highlights another area of future work: there is a need to create and share a reliable, flexible, cloud-based architecture that researchers can use to conduct studies using [shared gaze visualizations]”.

In this article, we present TittaLSL, a toolbox for real-time streaming of gaze and other data streams from a wide array of Tobii screen-based eye trackers^1^ in MATLAB and Python. TittaLSL is written making use of the powerful and widely supported Lab Streaming Layer (LSL) framework (Kothe et al., 2024), which provides robust methods for handling some of the hard parts of networked data sources, such as transparent discovery of remote data sources, synchronization and robust low-latency data transmission. However, while a researcher could use a general-purpose framework like LSL themselves to stream and receive eye-tracking data over the network, this would entail significant effort on their part and add significant complexity to their code. They would have to, among other things, convert eye tracker data to a format LSL accepts and to convert it back on the receiving side, while maintaining low latency. TittaLSL takes away this complexity for the end user. It builds on top of LSL to enable low-latency sending and receiving eye tracker data from Tobii eye trackers in a plug and play fashion requiring only a few lines of code. TittaLSL is designed to have a programming interface (API) and data format as similar as possible to the Titta toolbox for interacting with locally connected Tobii eye trackers (Niehorster et al., 2020a), meaning that local and remote eye tracker data streams can be handled in a uniform fashion. It should be noted, however, that none of TittaLSL’s functionality depends on Titta and that TittaLSL can thus be used without Titta.

TittaLSL enables straightforward implementation of multiple classes of experiments and also experiment management tools. Besides experiments where gaze data must be streamed at low latency to enable, for instance, shared gaze displays (e.g., Brennan et al., 2008; Niehorster et al., 2019; D’Angelo & Schneider, 2021), TittaLSL can also more generally simplify conducting experiments adopting a hyperscanning approach where multiple participants are eye tracked to study phenomena such as joint attention (Yu & Smith, 2017, 2013; D’Angelo & Begel, 2017) and interpersonal communication (Capozzi & Ristic, 2022; Mayrand et al., 2023). Whereas the latter class of studies can be conducted using independent data recordings that are synchronized afterwards during analysis, using TittaLSL this synchronization problem can be avoided by directly recording synchronized gaze data streams from all the eye trackers involved in the study. Similarly, since a plethora of devices other than eye trackers also support LSL (e.g., EEG, EMG, and fNIRS systems), TittaLSL makes it possible to construct setups where gaze data are recorded synchronized with any of these devices using, for instance, generic LSL recording tools such as its included LabRecorder tool. Finally, making the gaze data from one or multiple eye trackers available over the network enables implementing remote monitoring tools that can give an operator real-time insight into, for instance, the quality of the eye-tracking data (Niehorster et al., 2018, 2020b, c; Holmqvist et al., 2012) that is being collected.

## Tool description

Two parallel versions of TittaLSL have been developed. One is implemented in MATLAB and uses a MATLAB extension (MEX) file for parts of its implementation. The other is implemented as a compiled Python extension that is available for Python 3.8 and higher from the pip package archive: pip install TittaLSLPy. The two versions of TittaLSL have a very similar programming interface (only adjusted for the specifics of each language where needed) and can be used interchangeably (i.e., a gaze data stream created by the Python version of TittaLSL can be received by the MATLAB version of TittaLSL and vice versa). Full documentation and a complete listing for the programming interface (API) for both versions of TittaLSL is provided in the readme.md file in the Titta distributions at https://github.com/dcnieho/Titta/tree/master/LSL_streamer.

Titta uses Lab Streaming Layer (LSL, Kothe et al., 2024) under the hood for its eye tracker data streams, which means that other tools such as LSL’s LabRecorder program can also be used instead of TittaLSL for receiving or storing data streams created by TittaLSL. LSL is a long-standing toolbox that has been used as the core communication protocol in various other tools (e.g., Razavi et al., 2022; Wang et al., 2023; Iwama et al., 2024). TittaLSL is available for Windows and Linux.

The functionality of TittaLSL is divided into two classes. A Sender class is used for making eye tracker data available on the network (this is known as an outlet in LSL terminology). In MATLAB, TittaLSL.Sender represents a sender, whereas in Python, the class is called TittaLSLPy.Sender. The other class, a Receiver (Ti-tta LSL.Receiver and TittaLSLPy.Receiver, respectively) is used for recording TittaLSL data streams that are made available on the network by a TittaLSL sender (these are known as inlets in LSL). A single Sender can send multiple types of data (e.g., Tobii’s gaze and positioning streams). To keep the implementation simple and performant, receivers, on the other hand, are tied to a specific data stream; as such two separate receivers should be used to receive the gaze and positioning streams from a single remote eye tracker, and one when receiving gaze data from multiple remote eye trackers, one receiver should be used per remote eye tracker. The function TittaLSL.Receiver.Get-Streams() is used to discover either all TittaLSL data streams on the network, or only streams of a specific type (such as gaze streams).

One of the design goals of TittaLSL was to make it possible to handle data streams from local and remote eye trackers in a uniform manner. The interface for receiving data from a remote eye tracker using TittaLSL therefore closely mirrors that of Titta’s for locally connected eye trackers. For instance, while a local gaze sample would be retrieved from Titta’s sample buffer using TittaMex.consumeN(’gaze’,1), a gaze sample from a remote eye tracker is retrieved using Receiver.consumeN(1), where Receiver is a previously constructed TittaLSL.Receiver connected to a remote gaze stream. Furthermore, the data structures used by Titta and TittaLSL for, e.g., a gaze sample, are identical, with the exception of the naming of timestamp field. A TittaLSL sample has both a local and a remote system time (see below). This uniform interface for local and remote eye tracker data and data streams makes it easy to adapt experiments using a local eye tracker to an eye tracker running on a different machine or to extend the experiment to a multi-participant paradigm. For instance, as shown in the examples mentioned below, already-written code for processing or displaying data from a local eye tracker can be used for remote eye trackers with minimal or no modification.

When a TittaLSL receiver is started, all received remote eye tracker data are stored in a buffer. Like Titta, the data in the buffer can be accessed in two ways: consuming and peeking. While a consuming access removes the returned data from the buffer, a peeking access does not, leaving the retrieved samples available for later access. Peeking access can for instance be used when the experiment paradigm requires online access to the remote eye tracker data but all data should also be stored at the end of the recording for later offline analysis. Using this access mode, it is straightforward to, for instance, show the gaze position of another participant in real time while ensuring that the buffer contains all remote gaze data that was received during the session.

Also like Titta, remote eye tracker data streams can be both peeked and consumed through two interfaces. Either a specific number of samples can be retrieved (Receiver.consumeN() and Receiver.peekN()) from the start or the end of the buffer, or all the samples between two given timestamps can be retrieved (Receiver

.consumeTimeRange() and Receiver.peekTime-Range()). Different from Titta, eye tracker data can be retrieved from a TittaLSL.Receiver using the time range interface both by specifying local timestamps and remote timestamps. Whereas remote timestamps are the timestamps for the data expressed in the remote computer’s clock, local timestamps represent the corresponding time in the receiving computer’s local clock. The synchronization between these two clocks required to transform remote timestamps to the local computer’s clock is performed by the time_correction interface provided by a LSL inlet (Kothe et al., 2024).

Importantly, all the sending, receiving and synchronizing of eye tracker data are handled in the background by TittaLSL, transparent to the user. As such, the user doesn’t have to intersperse their experiment code with complicated logic for sending or receiving eye tracker data over the network, nor do they have to write advanced and error-prone multi-threading logic for dealing with these tasks. It should also be noted here that such threading is not possible at all in MATLAB, while in Python (before version 3.13), it would not be possible for such code to run in parallel to the experiment logic (Beazley, 2015). This may cause unexpected stalls in either the experiment or the sending and receiving of eye tracker data, potentially causing data loss. TittaLSL avoids these complications and allows to safely send and receive eye-tracking data in parallel with just a few lines of code.

**Fig. 1:**
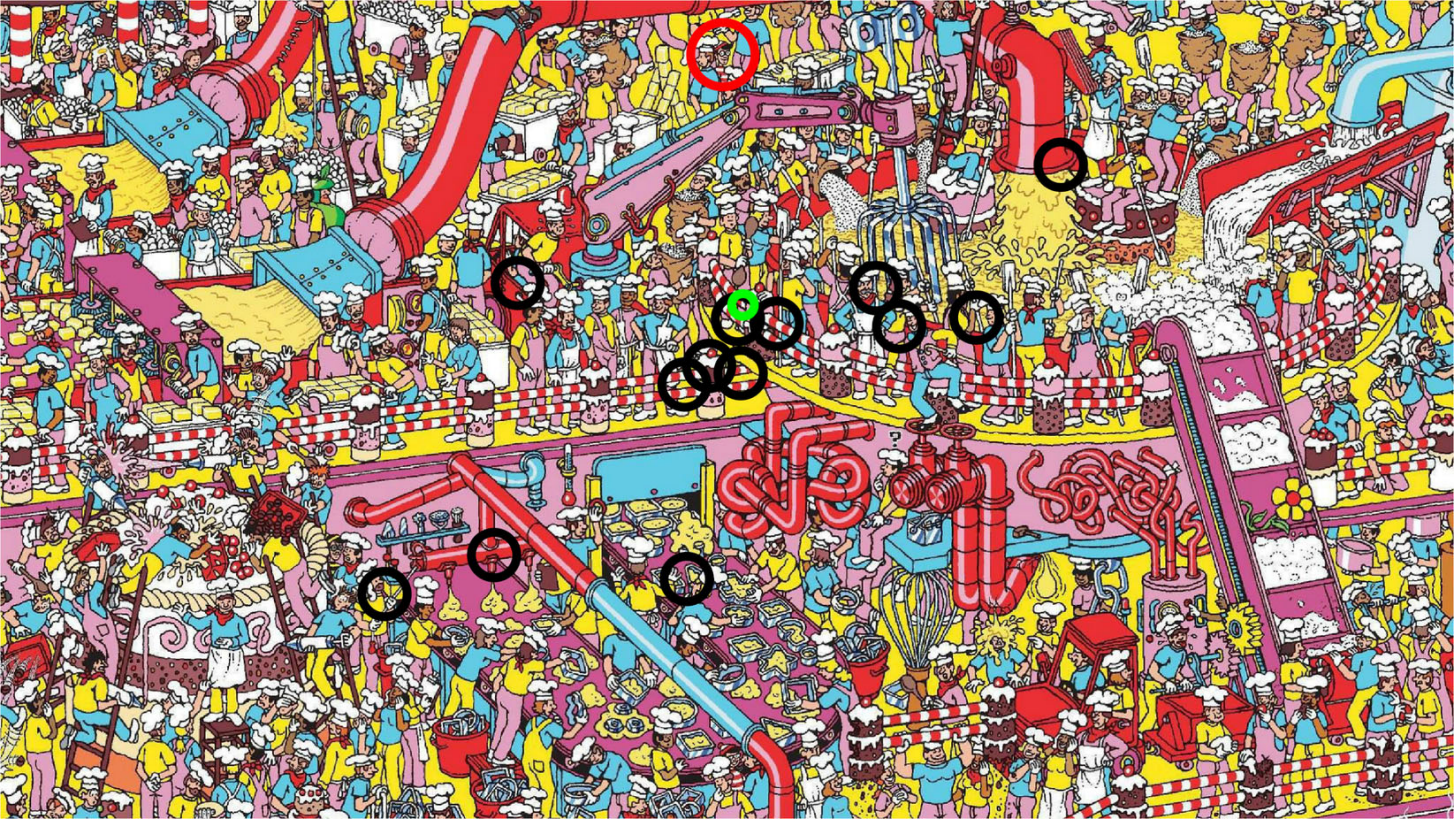
Data from an example run of the Where’s Waldo? demo after 1 s of search time has elapsed. Fourteen participants searched together for Waldo (indicated with the *red circle*–not visible during the experiment). Gaze data from the participant from whom this snapshot was taken is shown with the *green circle* while the gaze positions for the other 13 participants were shown to this participant using *black circles*

## Example use and getting started

TittaLSL has been designed to enable adding remote eye tracker data streams to existing experiments with minimal code modifications. The below steps show how to get started with TittaLSL.

1. For MATLAB, download TittaLSL from https://github.com/dcnieho/Titta/tree/master/LSL_streamer. If you are using MATLAB, it is already included with the Titta repository. For Python, acquire TittaLSL by installing the TittaLSLPy package. This can, for instance, be done by issuing pip install TittaLSLPy in the terminal.
2. Try out TittaLSL by running the scripts in the https://github.com/dcnieho/Titta/tree/master/LSL_streamer/demo_experiments folder for MATLAB, or the read_me_LSL.py and read_me_LSL_master.py scripts at https://github.com/marcus-nystrom/Titta/tree/master/demo_experiments/LSL_streamer for Python. These scripts implement a brief experiment where multiple participants can together search for Waldo in a highly crowded image (c.f., Nyström et al., 2017). While searching, the participant’s own gaze location as acquired with Titta is drawn on the screen, along with the gaze location of any other participants provided by TittaLSL remote gaze streams. An example is shown in Fig. 1. Note that a Python master can be combined with a MATLAB client, or vice versa.
3. To add TittaLSL to an existing experiment, copy over the required calls from the demos. Alternatively, the demos may provide a good starting point for developing a new experiment using data from a remote eye tracker. Compared to an experiment that connects only to a local eye tracker, the following additional steps are typically needed to add support for remote gaze streams using TittaLSL.
  - To make gaze data from a local eye tracker available on the network:
    i. Construct a TittaLSL.Sender() for the eye tracker connected locally to the computer. Below, the constructed sender is referred to as sender.
    ii. Call sender.create(<stream_type>) to create a source of eye tracker data that can be read remotely. The likely stream_type to use would be gaze, but the stream types externalSignal (for the eye tracker’s TTL port, if it has one), timeSync (for information about the eye-tracking device’s synchronization to the clock of the computer it is connected to) and positioning (for the eye tracker’s positioning stream) are also supported. Furthermore, if supported by the eye tracker, its eye openness signal (see Nyström et al., 2024) can be included in the provided gaze stream by calling sender.setIncludeEyeOpennessIn-Gaze().
    iii. By default, creating a data stream immediately starts streaming the data over the network as well. It is, however, also possible to manually start and stop a data stream using sender.start(<stream_type>) and sender.stop(<stream_type>), respectively.
    iv. Finally, once a data stream is no longer needed, it can be completely cleaned up using sender.destroy(<stream_type>).
  - To receive remote eye tracker data over the network:
    1. First discover what remote streams are available on the network using TittaLSL.Receiver.GetStreams(<stream_type>). The likely stream_type to use would be gaze, but the stream types externalSignal, timeSync and positioning (see above) are also supported.
    2. Construct a TittaLSL.Receiver() for a discovered stream. Below, the constructed re-ceiver is referred to as receiver.
    3. By default, creating a data stream receiver immediately starts reception of eye tracker data over the network. It is, however, also possible to manually start and stop receiving from a data stream using receiver.start() and receiver.stop(), respectively.
    4. As described in the “Tool description” section above, the received data can be consumed (data are removed from the buffer) using the receiver.consumeN() and receiver. consumeTimeRange() pair of functions, or peeked (data are kept in the buffer) using the receiver.peekN() and receiver. peekTimeRange() pair of functions. Data can furthermore be cleared from the receive buffer without reading it using the receiver.clear() and receiver.clearTimeRa-nge() functions.
    5. Finally, once a data stream receiver and the data in its buffer are no longer needed, it can be cleaned up by destructing the receiver object (e.g., using delete in MATLAB).

Documentation detailing the complete interface of TittaLSL is available in the readme.md file in the Titta distribution at https://github.com/dcnieho/Titta/tree/master/LSL_streamer.

It should be noted that since LSL (Kothe et al., 2024), the library on which TittaLSL is built, only supports local area networks (LANs), TittaLSL is not suitable for use over the internet without using advanced network configurations such as virtual private networks (VPNs). Important for such uses, as well as use over local connections with higher latency or lower reliability such as wireless links (see Nyström et al., 2017), is that the underlying LSL library implements several techniques to ensure reliable transmission without data loss (Kothe et al., 2024).

## Evaluation

A design goal for TittaLSL was to enable real-time gaze-sharing experiments using Tobii eye trackers. To meet this goal, it is essential that the latency of the gaze stream is minimal. Here we therefore evaluate the latency of a remote gaze stream received through TittaLSL and compare it to the latency of receiving the same gaze stream locally on the computer that the eye tracker is connected to.

Specifically, for this test, we implemented a script in Python (available here: https://github.com/marcus-nystrom/Titta/tree/master/demo_experiments/LSL_streamer/LSL_latency_test.py) that performed the following steps:

1. Upon startup, the script uses Titta to connect to the local eye tracker, and also starts an LSL Sender for this eye tracker’s gaze stream (which consists of 43 channels represented as doubles sent at 600 Hz, resulting in an outgoing data rate of about 200 kilobytes/s, excluding overhead, per listener).
2. For up to 100 s, the script then searches for other TittaLSL sources of gaze data on the network, and opens a TittaLSLPy.Receiver to connect to the remote gaze stream. This phase may end earlier when connections have been established with a predefined number of other clients.
3. Once connected to enough clients or after 100 s, the script starts sending gaze through the LSL Sender for one minute. Simultaneously, a receiver process is started per stream to timestamp the arrival of both the local and the remote gaze samples. Each receiver process, for both the local and the remote streams, did the following. It continuously polled the gaze buffer of Titta or TittaLSL for new samples, and timestamped each new sample as it was received. Since the timestamp of a gaze sample delivered by the Tobii eye tracker reflects the moment the camera image was taken from which the gaze sample derives, this timestamp when data was received allows judging the end-to-end latency from the moment of sample acquisition until the moment the sample is available to the Python script using the calculation $t_{receive}-t_{Tobii}$ where $t_{receive}$ is the timestamp assigned to a sample when it was received by our script, and $t_{Tobii}$ the sample’s timestamp provided by Tobii. For remote streams, the sample’s timestamp transformed to the receiving computer’s clock, as provided by TittaLSL for each sample, was used. One separate process per client was used because of the inability of the version of Python we used (3.10) to perform parallel processing using multithreading (Beazley, 2015), which would limit our ability to accurately timestamp when samples arrive for a large number of data streams.

**Fig. 2:**
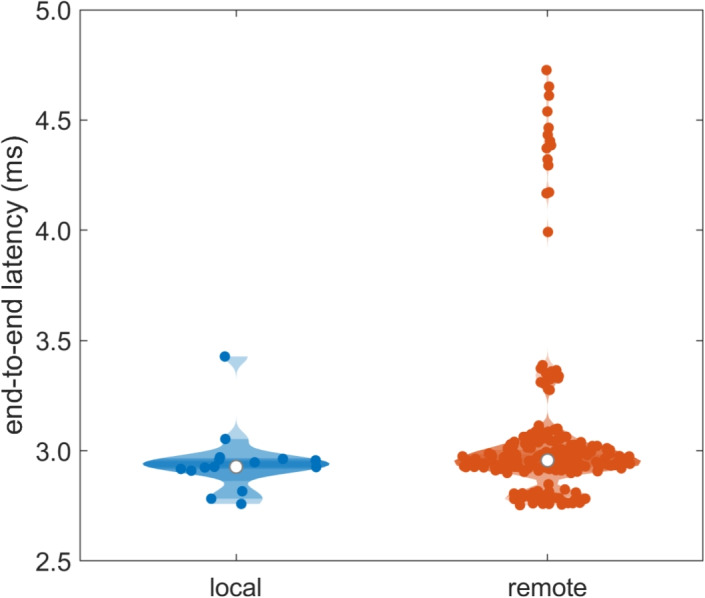
Mean end-to-end latencies for receiving gaze data from the locally connected eye tracker and for receiving remote gaze data via TittaLSL from eye trackers connected to other stations. As can be seen, the distribution of remote latencies is nearly identical to the latency distribution for receiving the gaze stream locally. The mean latencies above 4 ms were all for receipt of the gaze stream sent from a single station. Many more data points are available for remote latencies than for local latencies as the data stream for each station was received by all the other stations in the setup

The test was performed with TittaPy and TittaLSLPy versions 1.4.2. Fifteen simultaneous eye-tracking streams were used in the digital classroom environment at the Lund University Humanities Lab. While this environment is described in detail in another publication (Niehorster et al., 2024a; see also Nyström et al., 2017), its main characteristics are described here. The digital classroom contains 15 separate student stations that are each equipped with a screen-based eye tracker (Tobii Pro Spectrum, firmware version 2.9.0-oculus-0) and a computer (equipped with an Intel Core i7-9800X processor, 32 GB of RAM and a Marvell FastLinQ QL41112HLRJ 10 Gb network card and running Windows server 2022). These computers are connected via two 10-GB links to an Aruba 8320 switch. Participants, who provided informed consent, were placed at the stations and were asked to look at the eye tracker’s screen while their gaze data were recorded at 600 Hz.

The mean latency for both local and remote gaze streams were computed per stream for the one minute of recorded data. The distribution of mean latencies is shown by means of violin plots in Fig. 2, which compares the latency for locally receiving a sample from the eye tracker (using Titta) and for receiving a sample from a remote eye tracker (via TittaLSL). As can be seen, latencies for remote samples (grand mean: 3.05 ms) are nearly identical to the latencies for receiving a sample from the eye tracker when it is locally connected to a station (grand mean: 2.95 ms). Furthermore, for each data stream, the jitter of the latencies was determined by means of the standard deviation. Jitter in the latencies was low for both local (grand mean 0.54 ms) and remote streams (grand mean 1.58 ms). It should be noted that the reported latencies are well below the refresh interval of even high refresh rate screens (e.g., 4.2 ms for a 240-Hz screen). This indicates that TittaLSL is a suitable solution even for experiments that require the gaze of participants to be shown to other participants with the minimal possible latency given the display system.

## Conclusion

In this article, we presented TittaLSL, a MATLAB and Python tool that provides a simple but powerful way of creating and using eye-tracking data streams over a local network. Given that the interface of TittaLSL is very similar to that of Titta (Niehorster et al., 2020a), we expect that TittaLSL has a low barrier to entry for adapting existing experiments to using networked eye trackers or for creating new networked eye-tracking experiments such as multi-participant paradigms (D’Angelo & Schneider, 2021; Wahn & Schmitz, 2023).

## Data Availability

TittaLSL is available as an extension to the Titta toolbox at https://github.com/dcnieho/Titta. This repository also contains MATLAB examples using the toolbox. Python examples are available from https://github.com/marcus-nystrom/Titta.

## Footnotes

1 At the time of writing, the following 14 models are supported: Tobii Pro Spectrum, Tobii Pro Fusion, Tobii Pro TX300, Tobii Pro T60 XL, Tobii Pro Spark, Tobii Pro Nano, Tobii Pro X3-120, Tobii Pro X2-60, Tobii Pro X2-30, Tobii Pro X60, Tobii Pro X120, Tobii Pro T60, Tobii Pro T120, Tobii 4C.

## Code availability

TittaLSL is available as an extension to the Titta toolbox at https://github.com/dcnieho/Titta. This repository also contains MATLAB examples using the toolbox. Python examples are available from https://github.com/marcus-nystrom/Titta.

## Declarations

### Open Practices Statement

TittaLSL is available as an extension to the Titta toolbox at https://github.com/dcnieho/Titta. This repository also contains MATLAB examples using the toolbox. Python examples are available from https://github.com/marcus-nystrom/Titta.
